# Response of Soil Protist Community to Grazing Disturbance in an Alpine Grassland on the Eastern Qinghai–Tibet Plateau

**DOI:** 10.3390/microorganisms14071555

**Published:** 2026-07-16

**Authors:** Caicai Sun, Haitao An, Quanmin Dong, Wenting Liu, Weidong Lv, Xiaoxia Yang, Zhaojun Wang

**Affiliations:** 1 College of Agriculture and Food Engineering, Wuwei Vocational and Technical University, Wuwei 733000, China; suncaicai@wwvtu.edu.cn (C.S.); anht@wwvtu.edu.cn (H.A.); 2 Academy of Animal Science and Veterinary Medicine, Qinghai University, Xining 810016, China; qmdong@qhu.edu.cn (Q.D.); qhdxlwt@163.com (W.L.); 18215358641@163.com (W.L.); 3 Qinghai Provincial Key Laboratory of Adaptive Management on Alpine Grassland, Xining 810016, China

**Keywords:** alpine grassland, grazing intensity, soil protist community

## Abstract

Grazing disturbance strongly affects grassland ecosystem structure and function, but its impacts on soil protist communities across vertical soil profiles remain unclear in alpine grasslands. We investigated soil protist community composition, alpha diversity, and their relationships with environmental factors under four grazing intensities (no grazing, light, moderate, heavy grazing) at three depths (0–10, 10–20, 20–30 cm) on the eastern Qinghai–Tibet Plateau. Our results showed that grazing significantly altered the relative abundance of dominant protist orders, including Pyrenomonadales, Kinetoplastida, and Amoebida. The Shannon–Wiener and Pielou indices of protists decreased significantly with increasing soil depth, while grazing mainly reduced the Margalef index. Soil total nitrogen, bulk density, and pH were the dominant factors shaping soil protist community structure. Overall, soil protists were more sensitive to soil depth than grazing intensity. These findings improve our understanding of vertical protist distribution under grazing disturbance and provide a scientific basis for sustainable grassland management.

## 1. Introduction

Soil is a specific layer of the Earth, known as the pedosphere (which includes the lower part of the atmosphere, the upper part of the hydrosphere, and a portion of the biosphere), and is exceptionally rich in diverse groups of living organisms. Soil organisms are key drivers of nutrient cycling in soil ecosystems and play an indispensable role in maintaining soil health [1]. Previous studies primarily focused on soil bacteria, soil fungi, and soil archaea. For example, the important roles of soil bacteria in ecosystems [2], the ecological functions of fungi as decomposers in ecosystems [3], and the adaptability of soil archaea in extreme environments [4] have all become current research hotspots. However, these studies mainly concentrated on specific soil biological groups, without considering the numerous groups of other soil inhabitants that exist in soil.

Compared to the above-mentioned soil organisms, soil protists, as a unique component of eukaryotes [5], have gradually gained attention. Soil protists are the most diverse eukaryotes apart from animals, plants and fungi, and they are widely distributed and play a significant role in ecosystems, both in terms of abundance (with tens of thousands or even more individuals in one gram of soil) [6] and their ecological service functions [7,8]. Unlike bacteria and fungi, soil protists occupy multiple trophic levels in ecosystems [9]. For instance, as primary producers and decomposers, they regulate the degradation of organic matter and carbon fixation, thereby promoting nutrient cycling processes [10]. Phototrophic protists, for example, contain pigments and chloroplasts, enabling them to assimilate carbon dioxide through photosynthesis and synthesize organic compounds such as glucose, which contribute significantly to soil organic matter inputs [11]. As consumers, protists stimulate the mineralization of carbon and phosphorus, playing a crucial role in plant nutrient absorption and soil nutrient cycling [12]. Predatory protists enhance soil nutrient cycling by feeding on bacteria and fungi. This is because, compared to the carbon-to-nitrogen ratio of microbial cells, protist cells have relatively higher carbon and nitrogen content. After predatory protists consume other microorganisms, they excrete excess nitrogen from their bodies in the form of ammonium, which enhances nitrogen mineralization in the soil and facilitates plant growth [13]. These unique ecological functions underscore the irreplaceable role of soil protists in maintaining ecosystem stability and functionality.

The Tibetan Plateau is the highest plateau in the world (with an average elevation above 4000 m), and its unique geographic environment makes the region an ecologically fragile area [14]. This study was conducted in the alpine grasslands of the eastern Tibetan Plateau, which represent typical and widely distributed zonal vegetation of the plateau and are highly sensitive to grazing disturbance. This region is therefore ideal for revealing the general responses of alpine grassland ecosystems to grazing pressure. As one of the most significant ecosystems on the plateau, alpine grasslands cover approximately 60% of its total area and have become a focal point for global research on alpine ecosystems [15]. These grasslands play a critical role in maintaining regional biodiversity and global ecosystem functions [16]. However, alpine grassland ecosystems are facing significant ecological pressures, among which grazing is considered the most critical and valuable way of grassland utilization [17]. Grazing livestock primarily exert significant direct effects on the surface soil environment through activities such as foraging and trampling [18], while indirect effects (e.g., nutrient return via dung and urine and nutrient cycling caused by vegetation changes) further influence the physicochemical properties of deeper soil layers [19]. For instance, the decline in vegetation cover caused by grazing could lead to reduced deep soil moisture, while nutrient infiltration from dung and urine return might alter the nutrient structure and biological community in deeper soil layers [20]. These impacts could result in changes in the vertical distribution and functions of the soil protist community [21], especially under varying grazing intensities. The observed shifts in soil protist communities along grazing and soil depth gradients indicate the early ecological responses of alpine grasslands to grazing disturbance. Such changes reflect the degradation status and functional stability of plateau grassland soils, providing important implications for sustainable grazing management and ecological protection on the Tibetan Plateau.

Compared to soil microorganisms such as bacteria and fungi, research on soil protists remains insufficient, particularly in grassland ecosystems [22]. Additionally, although previous studies indicated that grazing significantly affected the diversity and distribution of soil biology community, most of these studies focused on surface soil, with relatively little attention given to the vertical distribution and functional changes of the soil protist community across different soil layers [23]. Deep soil plays a critical role in nutrient cycling and carbon storage, and neglecting its study may lead to a fragmented understanding of ecosystem functions. Exploring the effects of grazing intensity on soil protists in different soil layers, as well as the complex interactions with the unique environmental conditions and ecological functions of alpine grasslands, is currently an underexplored but highly significant research direction in alpine grassland soil ecology. Recent studies have revealed the impacts of grazing on soil protist communities in grasslands. For example, long-term grazing significantly alters soil protist community composition in desert steppes [24]. Grazing intensity can also regulate soil microbial network complexity and predator–prey relationships involving protists [25]. Moreover, heavy grazing tends to reduce belowground biodiversity and increase community dominance, further affecting soil biota structure [26]. These findings highlight the importance of grazing impacts on soil protists across vertical soil profiles. Such exploration helps to fill the knowledge gap between belowground biodiversity and ecosystem function responses, providing scientific evidence for the sustainable management of alpine grasslands. To fill this research gap, we conducted a manipulated grazing treatment with different grazing intensities in an alpine grassland around Qinghai Lake on the Qinghai–Tibet Plateau. Using high-throughput sequencing technology, we analyzed the community composition of soil protists and sought to answer the following questions: (1) What are the dominant components of the soil protist community in alpine grasslands? (2) Does the diversity of the soil protist community change with different grazing intensities and soil depths? (3) Through what mechanisms does grazing regulate the soil protist community? This study would enhance the understanding of the effects of grazing on the soil protist community across different soil layers in alpine grasslands and provides a scientific basis for ecological monitoring and grassland management in the alpine grasslands of the Qinghai–Tibet Plateau.

## 2. Materials and Methods

### 2.1. Experimental Site

This experiment is conducted based on the “Qinghai Provincial Alpine Grassland-Livestock Adaptive Management Technology Platform,” which is located in Xihai Town, Haiyan County, Haibei Tibetan Autonomous Prefecture, Qinghai Province, China (36°44′–37°39′ N, 100°23′–101°20′ E, at an altitude of 3150 m). The platform is managed by the Qinghai Provincial Department of Science and Technology. This region experiences a plateau continental climate, with an average annual temperature of 1.5 °C. During the non-growing season, the temperature drops to −24.8 °C, while in the growing season, it reaches 12.5 °C. Annual precipitation averages 400 mm, primarily occurring between June and September. The soil in the study area is classified as alpine meadow soil with a sandy loam texture, and the grassland type is alpine meadow, predominantly composed of species such as *Stipa purpurea*, *Poa annua*, *Kobresia humilis*, *Carex aridula*, and *Potentilla acaulis* [27,28].

### 2.2. Experimental Design

**Figure 1:**
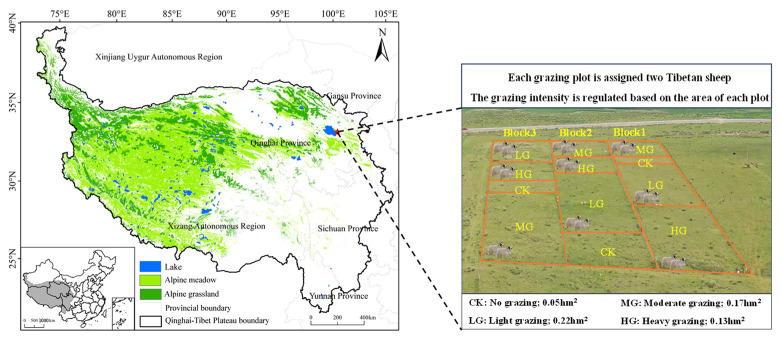
Geographical locations of the experimental site and the layout of grazing intensity experimental plots.

In 2018, alpine grassland as an experimental area of flat terrain with relatively uniform environmental and vegetation conditions was selected and fenced to exclude livestock grazing (before setting up the enclosures, we conducted a vegetation survey to ensure that the grassland conditions were relatively uniform). A randomized block design was implemented, with four grazing intensity treatments established: no grazing (CK, plot area: 0.05 hm^2^, stocking rate: 0 Tibetan sheep·hm^−2^, grassland utilization rate: 0%), light grazing (LG, plot area: 0.22 hm^2^, stocking rate: 3.03 Tibetan sheep·hm^−2^, grassland utilization rate: 30–35%), moderate grazing (MG, plot area: 0.17 hm^2^, stocking rate: 3.86 Tibetan sheep·hm^−2^, grassland utilization rate: 50–55%), and heavy grazing (HG, plot area: 0.13 hm^2^, stocking rate: 5.13 Tibetan sheep·hm^−2^, grassland utilization rate: 65–70%). Each grazing intensity treatment included three replicates, resulting in a total of 12 grazing plots, each separated by fencing (Figure 1). Plot areas differed among grazing treatments to achieve targeted stocking rates and utilization rates, as illustrated in Figure 1. Tibetan sheep, a unique livestock species on the Tibetan Plateau, were used for grazing. Two male Tibetan sheep (30 ± 2 kg, 1 year old) grazed each plot, with grazing intensity controlled by adjusting plot size. Grazing occurred from June to October annually, with monthly grazing time dynamically adjusted based on grassland aboveground biomass and the forage intake of livestock, typically lasting approximately 10 days per month. Livestock were not provided supplemental feed during the grazing period but had access to sufficient drinking water [29].

### 2.3. Sample Collection and Measurement

Plant and soil sampling were conducted concurrently in late July 2022. Within each grazing plot, three 0.5 × 0.5 m quadrats were randomly selected. Plants within each quadrat were cut at ground level, killed at 105 °C for one hour, and then dried at 70 °C to a constant weight to measure the aboveground biomass (AGB). A soil auger with a diameter of 7 cm was used to collect samples from the 0–10 cm, 10–20 cm, and 20–30 cm soil layers within each quadrat. Plant root samples were extracted from the collected soil, rinsed clean, and dried to a constant weight to determine the belowground biomass (BGB) [30].

Soil samples were collected from the same soil layers (0–10 cm, 10–20 cm, and 20–30 cm) using a soil auger with a diameter of 3.5 cm through random multi-point sampling (30 sampling points within each grazing plot). Soil samples from each plot and soil layer were thoroughly mixed, and impurities such as roots and stones were removed, resulting in a total of 36 composite samples (there were four grazing treatments, with three replicates for each treatment, totaling 12 grazing plots, and each grazing plot had three soil layers). Each composite sample was divided into two portions: one was stored at −80 °C for high-throughput sequencing of soil protists, and the other was stored at 4 °C for the determination of soil physicochemical properties.

Total DNA was extracted from soil samples using a Soil DNA Kit (Magen Biotechnology, Guangzhou, China), following the manufacturer’s protocols. Whole-metagenome high-throughput sequencing was performed on the Illumina NovaSeq platform (Illumina Inc., San Diego, CA, USA) to obtain raw sequencing data of mixed soil microorganisms, including bacteria, fungi, archaea, protists, and soil fauna. Unique barcodes were assigned to each sample during library construction. Sequencing libraries were constructed using the NEB Next^®^ Ultra DNA Library Prep Kit (New England Biolabs, Ipswich, MA, USA) for Illumina. Protist sequences were further bioinformatically extracted from the total metagenomic dataset via taxonomic annotation. Only protist-assigned sequences were retained for downstream community analyses. Taxonomic classification was performed against the Protist Ribosomal Reference (PR2) database (https://pr2-database.org/ (accessed on 20 June 2026)) [31]. Non-protist sequences (including fungi, metazoans, Rhodophyta, and Streptophyta) were filtered out to generate the final protist community matrix. Taxonomic orders with an average relative abundance > 1% were defined as dominant orders. Raw whole-metagenomic sequencing data generated in this study have been deposited in the NCBI SRA database under BioProject accession SUB16186799 (PRJNA accession pending). Protist sequences were extracted from shotgun metagenomic data following standard soil metagenomic workflows, with detailed parameters provided in the Supplementary Materials (Table S1). High-quality clean reads were obtained with sufficient sequencing depth to characterize the protist community composition.

Soil physicochemical properties were determined according to standard procedures described by Bao [32]. Soil moisture (SM) was measured by the oven-drying method at 105 °C for 24 h and calculated as the percentage mass loss after drying. Soil bulk density (BD) was determined using the core method, calculated as the ratio of soil dry weight to the volume of the soil core. Soil total carbon (TC) and total nitrogen (TN) were analyzed using a Vario TOC Select Analyzer (Elementar GmbH, Frankfurt, Germany) after fine grinding and sieving. Soil pH was measured in a 1:2.5 (soil:water) suspension using a pH 400 pH meter (Spectrum Technologies, Anaheim, CA, USA).

### 2.4. Data Analysis

#### 2.4.1. Calculation of Soil Protist Community Diversity

Alpha-diversity indices (Shannon–Wiener, Margalef, Pielou) were calculated at the order level using the vegan package (v2.6-4) in R software (version 4.3.1). Due to the nature of whole-metagenomic sequencing data, protist sequences were bioinformatically extracted from mixed microbial datasets, and reliable species-level (OTU/ASV) taxonomic assignment was not feasible. Therefore, order-level community analysis was adopted and order-level classification was used due to the low abundance of protists in shotgun metagenomic data and incomplete reference databases, which prevent reliable finer-scale taxonomic assignment, which is an acceptable approach for metagenome-derived protist community studies. The Shannon–Wiener index provided an integrated description of community diversity and was commonly used to evaluate ecosystem health. The Margalef index emphasized species richness, while the Pielou index focused on species evenness, with less influence from the total number of species. The calculation formulas for these indices are as follows: Shannon–Wiener index (*H* = −$\sum P_{i}ln(P_{i})$), Margalef index (*D* = (*S* − 1)/ln*N*), and Pielou index (*J* = H/ln*S*) were used to represent community diversity of soil protists in these formulas; *N* represents the total number of soil protists; *S* represents the number of orders; $P_{i}$ is the proportion of the number of individuals of order *i* to the total number of individuals in the soil protist community. Raw relative abundances were used for order-level protist community analysis to facilitate straightforward ecological interpretation of grazing-induced changes. The potential statistical limitation of compositional data without centered log-ratio transformation is acknowledged in the Section 4.

#### 2.4.2. Statistical Analysis

Statistical analyses were performed using R software (version 4.3.1, R Foundation for Statistical Computing, Vienna, Austria, 2023). Graphical representations were generated with Sigmaplot 14 software (Systat Software, San Jose, CA, USA). Normality was tested using the Shapiro–Wilk test, and homogeneity of variances was assessed using Levene’s test in R. A separate one-way ANOVA was used to test the independent effects of grazing intensity and soil depth on plant biomass, soil physicochemical properties, and soil protist community characteristics, respectively. Post hoc multiple comparisons were conducted using Tukey’s HSD test to correct for multiple testing. Beta-diversity was calculated based on Bray–Curtis distance matrices using the vegan package in R. Permutational multivariate analysis of variance (PERMANOVA, 999 permutations) was used to test whether grazing intensity and soil depth significantly altered protist community composition. To investigate the relationship between soil protists, plant biomass, and soil physicochemical properties, we applied redundancy analysis (RDA) to associate the composition of soil protist species with environmental variables, revealing the correlation between soil protist community ecological characteristics and environmental factors. We calculated the incremental explanatory power of variables using hierarchical partitioning implemented via the rdacca.hp R package (v1.1-7) [33] to quantify the independent contribution of different environmental factors to the soil protist community. Additionally, we constructed a structural equation model (SEM) via the piecewiseSEM R package (v2.3.0) in R software (version 4.3.1) to further elucidate the intrinsic relationships between the soil protist community and environmental factors [34].

## 3. Results

### 3.1. Effects of Grazing Intensity on Plant Biomass and Soil Physicochemical Properties

**Figure 2:**
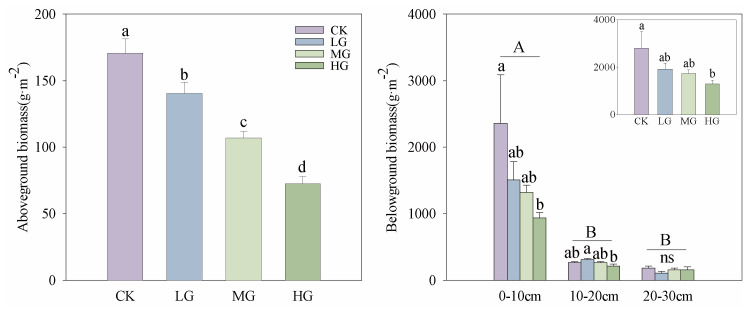
The effects of grazing intensity on plant aboveground and belowground biomass and the distribution of belowground biomass across soil layers. Note: in the figure, lowercase letters indicate significant differences among different grazing intensity treatments within the same soil layer; uppercase letters indicate significant differences between different soil layers without considering treatment factors; ns represents no significant difference (*p* > 0.05); the diagram in the top right corner shows significant differences among different grazing intensity treatments without considering soil layer factors (*p* < 0.05).

Aboveground plant biomass significantly decreased with increasing grazing intensity (*p* < 0.05). Belowground plant biomass showed a decreasing trend as grazing intensity increased, and compared to the CK treatment, the HG treatment significantly reduced belowground biomass by 53.50% (*p* < 0.05) (Figure 2, top right). Additionally, belowground biomass in the 0–10 cm soil layer was significantly higher than that in the 10–30 cm layer, accounting for 78.57% of the total belowground biomass in the 0–30 cm soil profile (Figure 2).

**Figure 3:**
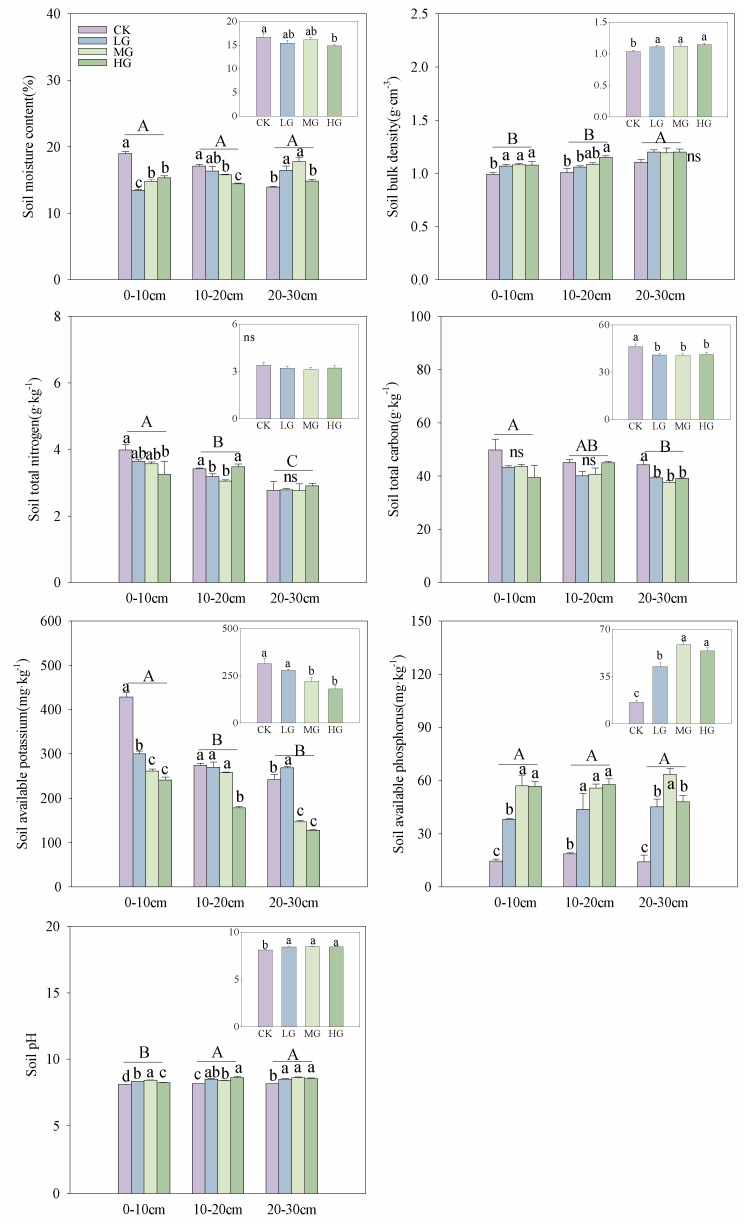
Effects of grazing intensity and soil depth on soil physicochemical properties. Insets show the overall effects of grazing intensity across all soil depths. in the figure, lowercase letters indicate significant differences among different grazing intensity treatments within the same soil layer; uppercase letters indicate significant differences between different soil layers without considering treatment factors; ns represents no significant difference (*p* > 0.05); the diagram in the top right corner shows significant differences among different grazing intensity treatments without considering soil layer factors (*p* < 0.05).

Compared to the CK treatment, the HG treatment significantly reduced soil water content (SM) by 10.97% (from 2809.21 to 1306.37, *p* < 0.05) (Figure 3, top right). Grazing significantly increased soil bulk density (BD), which reached its maximum (1.14) in the 20–30 cm layer, significantly higher than in the 0–10 cm (1.06) and 10–20 cm (1.08) layers (*p* < 0.05). Grazing had no significant effect on total nitrogen (TN), but TN differed significantly with depth (*p* < 0.05), showing the highest value (3.61) from 0–10 cm and the lowest (2.81) from 20–30 cm. Compared with CK (46.37), grazing significantly decreased total carbon (TC) to 41.19 in HG (*p* < 0.05). The MG and HG treatments significantly reduced available potassium by 29.54% and 42.12% relative to CK, respectively, with the highest available potassium (15.64) from 0–10 cm and the lowest (14.85) from 20–30 cm (*p* < 0.05). Grazing significantly increased available phosphorus (*p* < 0.05). In addition, grazing significantly increased soil pH, which was lowest (8.27) from 0–10 cm and highest (8.45) from 20–30 cm (*p* < 0.05) (Figure 3).

### 3.2. Effects of Grazing Intensity on Soil Protist Community Composition and Diversity

In this study, soil protists were classified into 11 orders, among which nine orders, including Kinetoplastida, Pyrenomonadales and Eucoccidiorida, were identified as dominant orders (Table 1). In the 0–10 cm soil layer, compared with the CK treatment, the LG treatment significantly reduced the relative abundance of Pyrenomonadales (*p* < 0.05). In the 10–20 cm soil layer, the LG treatment significantly increased the relative abundance of Prostomatida to the highest level but significantly reduced the relative abundance of Kinetoplastida and Pyrenomonadales (*p* < 0.05), while the HG treatment had no significant effect on them. In the 20–30 cm soil layer, the LG treatment increased the relative abundance of Pyrenomonadales.

**Figure 4:**
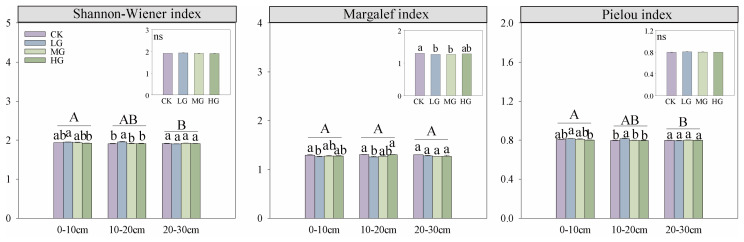
The effects of grazing intensity and soil depth on the diversity of soil protist community. in the figure, lowercase letters indicate significant differences among different grazing intensity treatments within the same soil layer; uppercase letters indicate significant differences between different soil layers without considering treatment factors; ns represents no significant difference (*p* > 0.05); the diagram in the top right corner shows significant differences among different grazing intensity treatments without considering soil layer factors (*p* < 0.05).

In the 0–10 cm soil layer, the LG treatment significantly reduced the Margalef index of soil protists compared to the CK treatment (*p* < 0.05). In the 10–20 cm soil layer, the LG treatment significantly increased both the Shannon–Wiener index and the Pielou index but significantly reduced the Margalef index compared to the CK treatment (*p* < 0.05). In the 20–30 cm soil layer, there were no significant differences in soil protist community diversity indices among different grazing intensity treatments. Overall, the Shannon–Wiener and Pielou indices of soil protists did not show significant differences across grazing treatments, and grazing reduced the Margalef index of soil protists (Figure 4, top right). The Shannon–Wiener and Pielou indices of soil protists decreased with increasing soil depth, with the highest values in the 0–10 cm soil layer (Figure 4).

**Figure 5:**
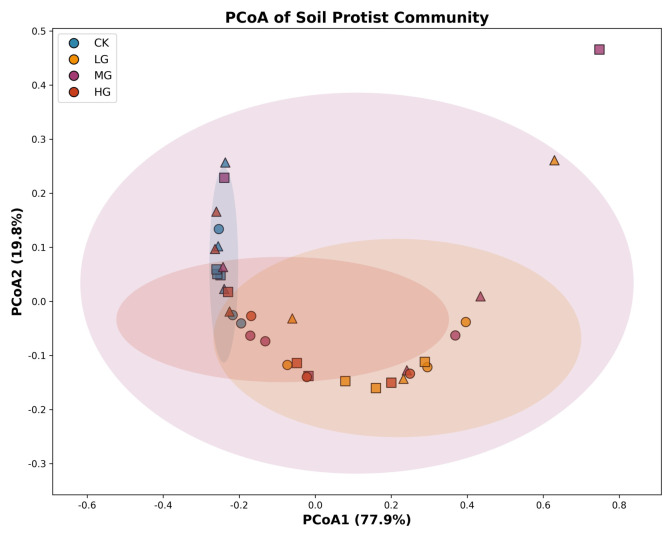
Principal coordinate analysis (PCoA) of soil protist community composition based on Bray–Curtis distance. Different colors represent different grazing intensities (CK, LG, MG, HG). Different shapes represent different soil depths. Ellipses indicate 95% confidence intervals.

Principal coordinate analysis (PCoA) based on Bray–Curtis distance showed that the first two principal axes explained 77.9% and 19.8% of the total variation in soil protist community composition, respectively, with a cumulative explanation rate of 97.7% (Figure 5). Permutation tests indicated that grazing intensity significantly altered the soil protist community composition (*p* < 0.001), whereas soil depth had no significant effect on community structure (*p* > 0.05). The PCoA ordination diagram clearly separated plots of different grazing intensities, further confirming that grazing intensity rather than soil depth was the key factor driving the shifts in soil protist community composition.

### 3.3. Relationships Between Soil Protist Community and Environmental Factors

**Figure 6:**
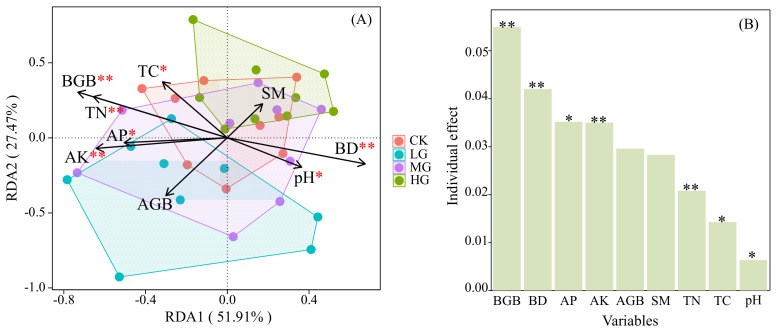
Redundancy Analysis (**A**) of Soil protist community and environmental Factors. Analysis of main environmental factors driving changes in soil protist community (**B**). AGB, Aboveground biomass; BGB, Belowground biomass; TN, Soil total nitrogen; TC, Soil total carbon; pH, Soil pH; BD, Soil bulk density; SM, Soil moisture content; AP, Soil available phosphorus; AK, Soil available potassium. * Represents *p* < 0.05, and ** Represents *p* < 0.01.

**Figure 7:**
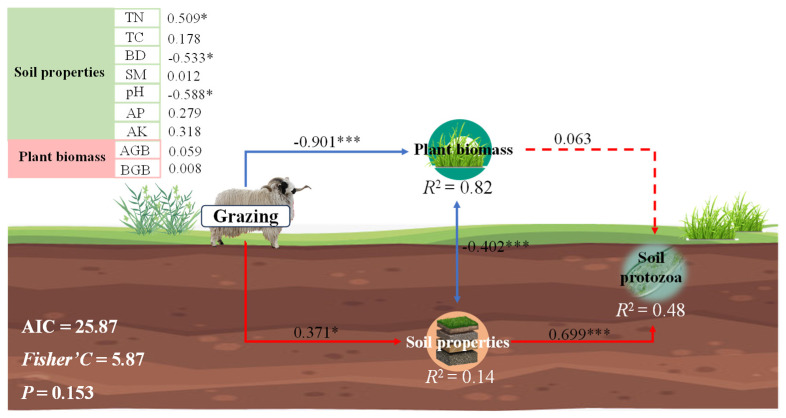
Piecewise structural equation model of the effects of grazing on soil protist communities. Red and blue arrows represent significant positive and negative effects, respectively, while solid and dashed lines indicate significant and non-significant effects, respectively. * Represents *p* < 0.05, and *** Represents *p* < 0.001.

To explore the relationship between environmental factors and the soil protist community, we conducted DCA analysis and found that the length of gradient was 0.070. Therefore, redundancy analysis (RDA) was applied. The results of the RDA (Figure 6) showed that the explanatory rates of the two axes were RDA1 = 51.91% and RDA2 = 27.47%. Belowground biomass, soil bulk density, available phosphorus, available potassium, total nitrogen, total carbon, and pH were significantly correlated with changes in the soil protist community (*p* < 0.05). The effects of these environmental factors on changes in the soil protist community were 20.64%, 15.79%, 13.23%, 13.16%, 7.82%, 5.38%, and 2.41%, respectively (Figure 6B). A structural equation model (SEM) was constructed to verify the intrinsic relationships between environmental factors and the soil protist community (Figure 7). The results showed that grazing had significant effects on plant biomass and soil characteristics, but soil characteristics were the primary correlates associated with soil protist community changes (path coefficient = 0.699, *p* < 0.001). Among these, total soil nitrogen, bulk density, and pH were the key soil characteristics that directly influenced the soil protist community (*p* < 0.05).

## 4. Discussion

### 4.1. The Impact of Grazing on Soil Protist Community

Soil protists are diverse unicellular eukaryotes characterized by their small size, simple structure, high diversity, widespread distribution, and short life cycles. They are highly sensitive to environmental disturbances and play a crucial role in maintaining microbiome stability and nutrient availability for plants, contributing significantly to ecosystem stability and balance [10]. Previous studies on soil organisms have primarily focused on soil bacteria and fungi, with relatively limited research on soil protists [35]. Independent studies across different grassland ecosystems have confirmed that grazing intensity strongly shapes soil protist community composition and trophic interactions. Consistent with findings in temperate and alpine grasslands, our results further support the generality of grazing–induced shifts in protist communities across regional scales [25,36,37]. This study explored the soil protist community under different grazing intensities on the eastern Qinghai–Tibet Plateau and found that the soil protists were primarily composed of Kinetoplastida, Pyrenomonadales, and Eucoccidiorida. These findings showed both similarities [38] and differences [22] compared to previous studies. The main reason for these differences was the high diversity of soil protists, as variations in regions (e.g., climate, altitude) and environments (e.g., soil conditions) led to significant differences in the composition of the soil protist community [37]. Specifically, the light grazing treatment significantly reduced the relative abundance of Pyrenomonadales, while moderate and heavy grazing treatments showed no significant changes (Table 1). Pyrenomonadales belongs to the phylum Cryptophyta, and most of its groups are autotrophic organisms. On the one hand, they utilize chloroplasts to convert light energy into chemical energy through photosynthesis to obtain the energy and nutrients necessary for their growth [39]. On the other hand, when light and oxygen are scarce, they can synthesize organic matter through chemotrophic processes for survival. The significant reduction in the relative abundance of Pyrenomonadales under light grazing might have been due to the slight disturbance of the plant community and minor changes in the soil environment, such as nutrient input and alterations in physical conditions [20], which were unfavorable for the survival of Pyrenomonadales. However, as grazing intensity increased to moderate or heavy levels, vegetation cover and biomass further declined, disturbances intensified, and livestock excreta input increased, potentially providing new nutrient sources and a suitable habitat for Pyrenomonadales, thereby maintaining their relative abundance. Additionally, the relative abundance of Amoeba significantly increased under light grazing, possibly associated with a more favorable environment and higher resource availability under light grazing. As heterotrophic organisms, Amoeba depend on external organic matter as a nutrient source; thus, an increased supply of organic matter could have provided ample food resources for them. However, as grazing intensity increased, despite the rise in livestock excreta, the intensified disturbance from grazing likely deteriorated the habitat conditions for Amoeba. As a result, moderate and heavy grazing did not lead to an increase in the relative abundance of Amoeba [40].

The Shannon–Wiener and Pielou indices of soil protists decreased with increasing soil depth (Figure 4). This phenomenon was primarily related to the environmental conditions of surface soil and deeper soil layers. Firstly, surface soils had higher belowground biomass and were rich in nutrients (Figure 2 and Figure 3), providing abundant food resources for soil protists [41]. In contrast, deep soils typically lacked organic matter and root exudates [42], resulting in resource scarcity and reduced protist species richness and diversity. Secondly, the bulk density of deeper soil was higher (Figure 2), reducing soil porosity and limiting oxygen and water content. Such conditions were unfavorable for the migration and survival of most soil protists [9], leading to a reduction in the Shannon–Wiener index of soil protists. Additionally, the surface soil environment of grazed alpine grasslands was influenced by various factors, including plant root systems, animal activities, and environmental changes, resulting in higher environmental complexity and resource diversity [43]. In contrast, deep soil environments were relatively stable and resource–poor but homogeneous [44], further restricting community diversity. Our study also revealed that grazing reduced the Margalef index of soil protists, particularly under light and moderate grazing treatments (Figure 4). The primary reason for this was that grazing compacted the soil, significantly increasing soil bulk density (Figure 2) and reducing microenvironments suitable for soil protists. Additionally, some protists, such as phototrophic soil protists, were highly sensitive to changes in soil structure and water availability [45], and their decline significantly reduced the Margalef index. However, under heavy grazing, the soil environment deteriorated, leading to a sharp decline or even disappearance of many sensitive protist species, simplifying the community structure. In this case, changes in the Margalef index were minimal, suggesting a potential shift toward a less diverse community state under heavy grazing.

### 4.2. The Relationship Between Soil Protist Community, Plant Biomass and Soil Physicochemical Properties

Soil total nitrogen, bulk density, and pH were key factors influencing the changes in soil protist community (Figure 7). Total nitrogen was also identified as a key environmental factor affecting the soil protist community, as reported in other studies [46]. The forms of nitrogen and other nutrients influenced the diversity of soil protists; for example, the abundance of some ciliates and algae was significantly affected by nitrogen gradients [47]. Furthermore, total nitrogen, as a critical resource for heterotrophic microorganisms (e.g., fungi and bacteria), indirectly promoted protist diversity, as these microorganisms served as essential food sources for protists [48]. However, soil microorganisms, including protists, were not only influenced by nitrogen but also significantly affected by other environmental factors, such as soil bulk density. In this study, soil bulk density had a significant negative effect on protist community. Grazing significantly increased soil bulk density (Figure 3), consistent with the findings of Stavi [49]. An increase in soil bulk density implies a reduction in the interstitial spaces between soil particles, leading to decreased soil aeration and the formation of a more compact soil environment, which makes it difficult for soil moisture to be effectively retained [50]. Many protists, such as Amoeba, rely on an aqueous film environment for movement and survival. Therefore, reduced soil moisture may be linked to limited habitat availability for protists. Additionally, higher soil bulk density implies restricted plant root growth, decreasing the number and activity of microorganisms in the soil. As microorganisms are potential prey for protists, reduced microbial abundance may be associated with lower protist survival. Consequently, there was a significant negative effect between protist community and soil bulk density. Furthermore, changes in soil bulk density can further alter microenvironmental factors, such as soil pH [51]. Most soil protists prefer to live in neutral or slightly acidic environments. However, in this study, with increasing grazing intensity, soil pH increased (Figure 3), leading to further alkalinization of the soil. This is detrimental to the survival of protists, causing some species to decline sharply or even disappear, resulting in a significant negative correlation between soil pH and protists. Cross–site comparisons and global syntheses have highlighted that soil nitrogen and pH are consistent key drivers of protist biogeography in grazed grasslands. Our observations align with these independent reports, reinforcing the broader applicability of our results beyond the Qinghai–Tibet Plateau region. In summary, based on long–term grazing experiments, grazing primarily affects the soil protist community by influencing soil characteristics. These findings enhance our understanding of the response of soil protist community changes to grazing and provide a scientific basis for ecological management of alpine grasslands. It should be emphasized that this observational study reveals statistical associations rather than causal relationships. The proposed mechanisms are hypotheses requiring further experimental validation.

## 5. Limitations of the Study

This study primarily investigated the impact of grazing on the soil protist community in alpine grasslands on the eastern Qinghai–Tibet Plateau. Relative abundance data are compositional, and untransformed Bray–Curtis distances were used here, consistent with standard soil protist community ecology practices. We identified the main components of the soil protist community in this region and explored the key environmental factors driving changes in these communities. However, as this study focused on sampling a specific alpine grassland site, it did not cover variations in the soil protist community across the broader Tibetan Plateau under different climatic conditions, soil types, and vegetation types. This limitation may restrict the generalizability of the study’s findings. In addition, our research was a cross–sectional study conducted at a specific time point, and thus it did not monitor the dynamic changes in the soil protist community under the long–term impact of grazing. Consequently, it was unable to reveal the seasonal or multi–year cumulative effects of grazing on protists. In the future, to address these limitations, subsequent studies should conduct sampling at a larger spatial scale, encompassing more diverse ecosystem types, to enhance the applicability and generality of the results. Furthermore, establishing long–term monitoring sites and conducting time–series studies will help explore the long–term effects and dynamic changes in soil protist communities and their functions under grazing. In the future, to address the above limitations, subsequent studies could conduct sampling on a larger spatial scale, covering more diverse ecosystem types, to improve the applicability and generalizability of the results. Additionally, long–term monitoring sites will be established, and time–series studies will be conducted to explore the long–term impacts of grazing on soil protist communities and their functional dynamics.

## 6. Conclusions

Our study showed that soil protists in alpine grassland pastures were mainly composed of Kinetoplastida, Eucoccidiorida and Pyrenomonadales. The soil protist communities responded differently to varying grazing intensities and, at the order level, Pyrenomonadales, Prostomatida, Amoebida and Kinetoplastida were more sensitive to grazing. Specifically, light grazing increased the relative abundance of Prostomatida and Amoebida but decreased the relative abundance of Pyrenomonadales and Kinetoplastida. Grazing reduced the Margalef index of soil protists, while the Shannon–Wiener index and Pielou index significantly decreased with increasing soil depth. Grazing indirectly influenced the soil protist community primarily by affecting soil characteristics, which in turn regulated the soil protist community. In conclusion, although our study indicated that the effects of different grazing intensities on the soil protist community were relatively minor, from a long–term perspective, excessive grazing intensity could potentially lead to the degradation of the soil protist community. Over time, this could impact the stability and sustainability of the alpine grassland ecosystem.

## Figures and Tables

**Table 1:** Impact of grazing intensity on the relative abundance of soil protists.

Soil Depth	Order	Treatments			
		CK	LG	MG	HG
0–10 cm	Diplomonadida	0.33 ± 0.04	0.37 ± 0.03	0.35 ± 0.03	0.31 ± 0.05
	Dictyosteliales	1.49 ± 0.04	1.53 ± 0.08	1.53 ± 0.11	1.38 ± 0.05
	Prostomatida	0.85 ± 0.03	0.88 ± 0.07	0.80 ± 0.08	0.72 ± 0.03
	Schizopyrenida	1.07 ± 0.03	1.05 ± 0.05	1.01 ± 0.04	0.99 ± 0.06
	Amoebida	10.57 ± 0.20	10.67 ± 0.05	10.40 ± 0.23	10.77 ± 0.21
	Kinetoplastida	28.62 ± 0.29	28.43 ± 0.20	29.24 ± 0.68	28.56 ± 0.24
	Haemosporida	4.05 ± 0.03	4.67 ± 0.35	4.65 ± 0.31	3.87 ± 0.08
	Piroplasmida	6.70 ± 0.08	6.77 ± 0.20	6.71 ± 0.06	6.59 ± 0.10
	Eucoccidiorida	13.58 ± 0.17	14.07 ± 0.13	13.43 ± 0.19	13.41 ± 0.30
	Cryptomonadales	13.01 ± 0.13 ^ab^	13.13 ± 0.10 ^ab^	12.80 ± 0.17 ^b^	13.34 ± 0.19 ^a^
	Pyrenomonadales	19.74 ± 0.24 ^a^	18.43 ± 0.17 ^b^	19.09 ± 0.43 ^ab^	20.06 ± 0.28 ^a^
10–20 cm	Diplomonadida	0.31 ± 0.04	0.31 ± 0.02	0.27 ± 0.01	0.31 ± 0.02
	Dictyosteliales	1.30 ± 0.04 ^ab^	1.37 ± 0.07 ^a^	1.17 ± 0.04 ^b^	1.23 ± 0.04 ^ab^
	Prostomatida	0.65 ± 0.04 ^b^	0.90 ± 0.02 ^a^	0.75 ± 0.07 ^ab^	0.75 ± 0.07 ^ab^
	Schizopyrenida	1.00 ± 0.09	1.18 ± 0.09	1.08 ± 0.07	1.04 ± 0.06
	Amoebida	9.90 ± 0.19 ^b^	10.54 ± 0.12 ^a^	10.17 ± 0.13 ^ab^	10.26 ± 0.08 ^ab^
	Kinetoplastida	29.93 ± 0.39 ^a^	28.02 ± 0.52 ^b^	30.04 ± 0.18 ^a^	29.73 ± 0.34 ^a^
	Haemosporida	4.13 ± 0.34	4.63 ± 0.13	4.29 ± 0.39	4.06 ± 0.17
	Piroplasmida	6.66 ± 0.08	7.68 ± 1.10	6.73 ± 0.05	6.49 ± 0.18
	Eucoccidiorida	14.16 ± 0.15	13.80 ± 0.16	14.41 ± 0.29	13.88 ± 0.11
	Cryptomonadales	12.39 ± 0.17 ^b^	13.17 ± 0.22 ^a^	12.55 ± 0.20 ^ab^	12.43 ± 0.21 ^b^
	Pyrenomonadales	19.57 ± 0.15 ^ab^	18.40 ± 0.53 ^c^	18.53 ± 0.27 ^bc^	19.81 ± 0.30 ^a^
20–30 cm	Diplomonadida	0.33 ± 0.02	0.32 ± 0.01	0.34 ± 0.03	0.28 ± 0.01
	Dictyosteliales	1.17 ± 0.04	1.10 ± 0.02	1.02 ± 0.09	1.12 ± 0.06
	Prostomatida	0.75 ± 0.03	0.68 ± 0.01	0.75 ± 0.04	0.78 ± 0.03
	Schizopyrenida	0.95 ± 0.04	0.96 ± 0.05	0.96 ± 0.07	1.08 ± 0.03
	Amoebida	9.96 ± 0.16	10.54 ± 0.22	10.31 ± 0.09	10.36 ± 0.24
	Kinetoplastida	29.62 ± 0.40	29.40 ± 0.52	29.13 ± 0.39	29.25 ± 0.25
	Haemosporida	4.36 ± 0.24	3.66 ± 0.21	4.36 ± 0.17	4.00 ± 0.18
	Piroplasmida	6.64 ± 0.12	6.60 ± 0.16	6.93 ± 0.24	6.59 ± 0.06
	Eucoccidiorida	14.02 ± 0.11	14.23 ± 0.24	14.69 ± 0.12	14.14 ± 0.43
	Cryptomonadales	12.46 ± 0.30	12.32 ± 0.29	12.77 ± 0.23	12.76 ± 0.10
	Pyrenomonadales	19.73 ± 0.19 ^ab^	20.18 ± 0.20 ^a^	19.65 ± 0.59 ^b^	19.57 ± 0.21 ^ab^

Note: Lowercase letters indicate significant differences in soil protist relative abundance between different treatments (*p* < 0.05).

## Data Availability

All raw whole-metagenomic sequencing data and matching BioSample metadata have been fully uploaded, processed and publicly released in the NCBI SRA database under BioProject accession PRJNA1490975. The temporary submission ID is SUB16303644, with official public release date on 7 July 2026. Permanent access link: https://www.ncbi.nlm.nih.gov/sra/PRJNA1490975. All FASTQ files are accessible and downloadable without any restrictions.

## Supplementary Materials

The following supporting information can be downloaded at: https://www.mdpi.com/article/10.3390/microorganisms14071555/s1, Table S1: Summary of shotgun metagenomic sequencing statistics for soil samples.
