# Supplementary material for: Response of Soil Protist Community to Grazing Disturbance in an Alpine Grassland on the Eastern Qinghai–Tibet Plateau
Source: Microorganisms. 2026 Jul 16;14(7):1555. doi: 10.3390/microorganisms14071555 (PMC13414387; doi:10.3390/microorganisms14071555)
Supplement: Supplementary file 1 [file microorganisms-14-01555-s001.zip › microorganisms-4290598-supplementary.pdf]

Table S1. Summary of shotgun metagenomic sequencing statistics for soil samples

Treatments	Soil layer	Rawreads	Cleanreads (%)	Adapter (%)	LowQuality (%)	polyA (%)	N (%)
CK	0-10cm	74179734	73994946 (99.75%)	17710 (0.02%)	164120 (0.22%)	0 (0.0%)	2958 (0.0%)
	0-10cm	70679362	70500672 (99.75%)	16558 (0.02%)	159644 (0.23%)	0 (0.0%)	2488 (0.0%)
	0-10cm	76311764	76120184 (99.75%)	18722 (0.02%)	169636 (0.22%)	0 (0.0%)	3222 (0.0%)
	10-20cm	71209424	71030954 (99.75%)	17350 (0.02%)	158318 (0.22%)	0 (0.0%)	2802 (0.0%)
	10-20cm	68020008	67824750 (99.71%)	16828 (0.02%)	175920 (0.26%)	0 (0.0%)	2510 (0.0%)
	10-20cm	76311376	76109074 (99.73%)	19880 (0.03%)	179070 (0.23%)	0 (0.0%)	3352 (0.0%)
	20-30cm	72544684	72360784 (99.75%)	19970 (0.03%)	161102 (0.22%)	0 (0.0%)	2828 (0.0%)
	20-30cm	71446796	71261420 (99.74%)	18130 (0.03%)	164356 (0.23%)	0 (0.0%)	2890 (0.0%)
	20-30cm	73504918	73315336 (99.74%)	19128 (0.03%)	167438 (0.23%)	0 (0.0%)	3016 (0.0%)
LG	0-10cm	85473286	85296620 (99.79%)	20192 (0.02%)	151760 (0.18%)	0 (0.0%)	4714 (0.01%)
	0-10cm	1.01E+08	100496470 (99.76%)	24168 (0.02%)	214830 (0.21%)	0 (0.0%)	5342 (0.01%)
	0-10cm	1.04E+08	103942948 (99.77%)	24052 (0.02%)	210458 (0.2%)	0 (0.0%)	5446 (0.01%)
	10-20cm	99068452	98840278 (99.77%)	23284 (0.02%)	199826 (0.2%)	0 (0.0%)	5064 (0.01%)
	10-20cm	1.24E+08	123918590 (99.69%)	40210 (0.03%)	335350 (0.27%)	0 (0.0%)	10070 (0.01%)
	10-20cm	87136228	86914616 (99.75%)	22778 (0.03%)	194046 (0.22%)	0 (0.0%)	4788 (0.01%)
	20-30cm	94464696	94256258 (99.78%)	22740 (0.02%)	180804 (0.19%)	0 (0.0%)	4894 (0.01%)
	20-30cm	1E+08	99885456 (99.78%)	25128 (0.03%)	192006 (0.19%)	0 (0.0%)	5534 (0.01%)
	20-30cm	90127092	89904076 (99.75%)	22946 (0.03%)	195356 (0.22%)	0 (0.0%)	4714 (0.01%)
MG	0-10cm	86228922	86010080 (99.75%)	22208 (0.03%)	192338 (0.22%)	0 (0.0%)	4296 (0.0%)
	0-10cm	80383156	80203554 (99.78%)	19114 (0.02%)	156542 (0.19%)	0 (0.0%)	3946 (0.0%)
	0-10cm	97039370	96818786 (99.77%)	22532 (0.02%)	193130 (0.2%)	0 (0.0%)	4922 (0.01%)
	10-20cm	82216586	82027940 (99.77%)	21300 (0.03%)	163310 (0.2%)	0 (0.0%)	4036 (0.0%)
	10-20cm	1.07E+08	10686324	26876	248502	0 (0.0%)	5496

			6 (99.74%)	(0.03%)	(0.23%)		(0.01%)
	10-20cm	95272292	95028990 (99.74%)	23948 (0.03%)	214728 (0.23%)	0 (0.0%)	4626 (0.0%)
	20-30cm	73871492	73682270 (99.74%)	20780 (0.03%)	164768 (0.22%)	0 (0.0%)	3674 (0.0%)
	20-30cm	1.25E+08	124557772 (99.75%)	30572 (0.02%)	272456 (0.22%)	0 (0.0%)	6272 (0.01%)
	20-30cm	87933070	87713778 (99.75%)	22114 (0.03%)	193032 (0.22%)	0 (0.0%)	4146 (0.0%)
	0-10cm	93863846	93664332 (99.79%)	21768 (0.02%)	173136 (0.18%)	0 (0.0%)	4610 (0.0%)
	0-10cm	77952580	77790386 (99.79%)	16550 (0.02%)	141920 (0.18%)	0 (0.0%)	3724 (0.0%)
	0-10cm	85791956	85618910 (99.80%)	19618 (0.02%)	149294 (0.17%)	0 (0.0%)	4134 (0.0%)
	10-20cm	79476886	79316138 (99.80%)	18372 (0.02%)	138536 (0.17%)	0 (0.0%)	3840 (0.0%)
HG	10-20cm	70163738	70012866 (99.78%)	16638 (0.02%)	130814 (0.19%)	0 (0.0%)	3420 (0.0%)
	10-20cm	75915212	75704790 (99.72%)	20446 (0.03%)	186546 (0.25%)	0 (0.0%)	3430 (0.0%)
	20-30cm	83966072	83759778 (99.75%)	20206 (0.02%)	181972 (0.22%)	0 (0.0%)	4116 (0.0%)
	20-30cm	90726348	90507054 (99.76%)	22584 (0.02%)	192162 (0.21%)	0 (0.0%)	4548 (0.01%)
	20-30cm	75527256	75335556 (99.75%)	19648 (0.03%)	169570 (0.22%)	0 (0.0%)	2482 (0.0%)
